# Menopause symptoms in women and its relation with using complementary and alternative medicines: A survey in southeast Iran

**DOI:** 10.3389/fpubh.2022.947061

**Published:** 2022-08-22

**Authors:** Mahlagha Dehghan, Zahra Isari, Mohammad Hossein Abbaszadeh, Asma Ghonchehpour

**Affiliations:** ^1^Department of Critical Care Nursing, Razi Faculty of Nursing and Midwifery, Kerman University of Medical Sciences, Kerman, Iran; ^2^Nursing Research Center, Kerman University of Medical Sciences, Kerman, Iran

**Keywords:** complementary and alternative medicine, menopause, women, symptom, psychological symptoms, somatic-vegetative symptoms, urogenital symptoms

## Abstract

**Background:**

Postmenopausal women are interested in using complementary and alternative medicine because of their menopausal symptoms and the side effects of chemical medications and hormone therapy. Therefore, this study aimed to investigate the relationship between the use of complementary medicine (CAM) and menopausal symptoms among postmenopausal women.

**Methods:**

This cross-sectional study was conducted among 288 postmenopausal women who were referred to health centers in Kerman, southeastern Iran, from 2020 to 2021. Data were collected using a demographic questionnaire, the CAM questionnaire, and the Menopause Rating Scale (MRS).

**Results:**

The mean score for the menopausal symptoms of the participants was 8.73 ± 6.11. Only 28.8% of the participants had no/little symptoms. About 65.3% of the participants used at least one type of CAM in the previous year. The most commonly used types of CAM were nutritional supplements, prayer, and medicinal herbs. A significant association was found between the use of medicinal herbs, dry cupping, relaxation and meditation, prayer, and menopausal symptoms. Women experiencing menopausal symptoms used more CAM methods than those without menopausal symptoms (Odds ratio = 2.25, 95% confidence interval = 1.33–3.80).

**Conclusion:**

The study results indicated that the severity of menopausal symptoms and scores in somatic-vegetative and urogenital domains were higher in CAM users compared to non-CAM users. But there was no significant difference in the psychological domain between CAM and non-CAM users. Based on the results, it is suggested that more research be done in different communities with different healthcare systems to find out how complementary and alternative medicine affects menopausal symptoms in women.

## Introduction

Menopause is defined as decreased ovarian follicular function and estrogen level, which may begin as early as your mid-30s or as late as your mid-50s. When a woman is in menopause, her menstrual period has been gone for 12 consecutive months (1). Today, 95% of the women in some communities are in menopause, and this population is predicted to reach 1.2 billion by 2030, with an annual increase of 47 million new cases (2). About five million Iranian women were in menopause in 2021 (3). However, menopause is affected by genetic factors, obesity, alcoholism, ethnicity, diet, vitamin D and calcium intake, prolonged menstrual periods, and the use of oral contraceptives (4). Therefore, 50–75% of women experience pre-menopausal or menopausal symptoms, including hot flashes and night sweats (vasomotor symptoms), insomnia, musculoskeletal pain, and urogenital disorders, such as incontinence, vaginal dryness, painful intercourse, anxiety, fatigue, and depression (5–7).

Hormone therapy and non-hormonal treatments, as well as CAM, are the ways to prevent and reduce menopausal symptoms (3, 8–10). Hormone therapy is the primary treatment for menopausal symptoms. However, many women cannot use this therapy due to an increased risk of endometrial and ovarian neoplasia, uterine cancer, breast cancer, ovarian cancer, and an increased risk of stroke (11, 12). Non-hormonal treatments, such as clonidine, gabapentin, and antidepressants, can also reduce menopausal symptoms, but they have many side effects, such as sleep disorders, dizziness, nausea, fatigue, and constipation (13). Therefore, women have focused on complementary and alternative medicine (CAM), which is more cost-effective and has fewer side effects than other therapies. People use complementary medicine to support and strengthen their general health, as well as to improve the symptoms of diseases or drug side effects (14).

CAM refers to a broad set of healthcare practices that are not part of the current healthcare system. According to recent studies, about 73.6% of women experiencing menopause in Turkey (15) and 70% of women with polycystic ovary syndrome (16) in Australia used CAM. Studies showed that dry and wet cuppings (17), acupuncture (18), acupressure (19), massage (20), relaxation (21) and yoga (22), aromatherapy (23), diets (24), homeopathy (25), music therapy (26), hydrotherapy (27), and prayer (28) affected the improvement of vasomotor symptoms, urogenital disorders, fatigue, and depression caused by menopause. In addition, herbal products, such as chamomile (29), Vitex agnus-castus (30), Hypericum Perforatum (31), Crocus sativus (32), Curcumin (33), Melissa Officinalis (34), Thymes (35), Wheat Germ (36), Fennel (Foeniculum vulgare) (37), Valerian Root (38), Ginger (39) and Flaxseed (40), and Soy Isoflavones (41) affected physical and psychological symptoms caused by menopause. Postmenopausal women increasingly use a variety of complementary medicine methods to improve their menopausal symptoms due to the widespread complications of drug and hormone therapies. No research in Iran examines the relationship between complementary medicine methods and menopausal symptoms. Therefore, this study aimed to investigate the relationship between menopausal symptoms and the use of complementary medicine methods among women who were referred to health centers in Kerman from 2020 to 2021.

## Methods

### Study design and setting

This cross-sectional study was conducted on postmenopausal women referred to health centers in Kerman from 2020 to 2021. Health centers provide healthcare for the community (42). Kerman is located in southeastern Iran and consists of 47 health centers, of which 31 centers collaborated with researchers. Only 10 health centers made the contact numbers of postmenopausal women available, and 21 health centers collaborated with researchers in face-to-face sampling.

### Sampling and sample size

The sample size was estimated to be 267 using Cochran's formula for an infinite population following the study's primary objective (*Z* = 1.96, *d* = 0.06). Power analysis calculations with G^*^Power software (version 3.1.9.2) indicated that (power = 90%, α = 0.05, number of groups, i.e., CAM users: yes/no = two) 86 participants in each group would be needed to detect an effect size of 0.5.

The convenience sampling method was used in the present study. Inclusion criteria were participants aged 50–60 years, in normal menopause (rather than by hysterectomy and surgery), and whose menstrual period had been gone for at least 12 consecutive months (19, 36). Incomplete questionnaires (say 10% or more) were excluded from the study. Totally 220 questionnaires were given to postmenopausal women referred to Kerman health centers. Of these, 189 were returned (a response rate of 85.9%) and 36 were excluded due to not being eligible. In the online sampling form, 191 questionnaires were completed (a response rate of 12.44%) and 47 questionnaires were excluded due to the inclusion criteria. After checking completed questionnaires by statistical analysis, 9 samples were excluded from the study. Therefore, 288 samples were included in the study. The overall response rate was 56.71%.

### Ethical issue

The Kerman University of Medical Sciences approved this project (IR.KMU.REC.1399.444 and NO: 99000487). Then, permission was issued to the management of the healthcare centers. The researcher provided some oral information, including the study goals and objectives, data confidentiality, and anonymity, and she assured the participants that they could withdraw from the study at any time. Then, written informed consent was obtained individually.

### Questionnaire

#### Demographic information questionnaire

This questionnaire includes the variables of age, education level, marital status, occupation, duration of menopause, number of children, a history of addiction (smoking), a history of diabetes, a history of ovarian and uterine cancer, a history of breast cancer, a history of hysterectomy, a history of hypertension, and a history of chronic diseases (43).

#### The menopause rating scale

Heinemann et al. designed the questionnaire, which is an international scale for assessing menopausal symptoms (44, 45). This scale consists of 11 items with three subscales of somatic-vegetative symptoms (hot flashes, night sweats, heart palpitations, sleep disorders, and muscle and joint disorders), psychological symptoms (depression, anxiety, fatigue, and irritability), and urogenital symptoms (sexual dysfunction, vaginal dryness, and urinary symptoms). This questionnaire was rated on a 5-point Likert scale (from zero: no symptoms to four: very severe) (44, 46). Scores for each subscale are based on the sum of the item scores of the respective subscale. The total score of the questionnaire is the sum of the subscale scores (45). The minimum score is zero, while the maximum is 44, with total scores ≤ 11, 12–35, and ≥ 36 being no symptoms, mild to moderate symptoms, and severe to very severe symptoms, respectively (47, 48). Heinemann confirmed the face validity and reliability of this questionnaire with a Cronbach's alpha coefficient of 0.98 and an intra-class correlation coefficient of 0.97 (48). In addition, Darsareh et al. (49) and Shakeri et al. (50) in Iran confirmed the validity of this scale. Masjoudi et al. (48) in Iran reported a Cronbach's alpha coefficient of 0.83 for this questionnaire. In the present study, Cronbach's alpha coefficient was 0.86.

#### CAM questionnaire and Satisfaction with the use of complementary medicine questionnaire

The questionnaire of Dehghan et al. was used to assess the use of complementary medicine among women in the menopausal stage. This questionnaire contains 10 items on the use of some types of CAM (herbal medicine, wet cupping, dry cupping, massage, acupuncture, homeopathy, and relaxation techniques, such as yoga and prayer). Yes-no questions are used; if the answer is yes, the rate of consumption will be asked (from rarely to once a day) (50). Nine questions were asked to measure satisfaction with the use of complementary drugs, including their accessibility, ease of use, harmlessness, non-interference with daily activities, concerns about drug interaction, feeling good after using the treatment, suggesting this method to others, and their inexpensiveness. This questionnaire is scored on a five-point Likert scale (from four = very satisfied to zero = no idea). The score for satisfaction with the use of complementary medicines ranged from 0 to 36, with high scores indicating high satisfaction. Dehghan et al. (51) confirmed the questionnaire reliability (0.96), while Sheikhrabori (52) confirmed its validity (0.77). In the present study, Cronbach's alpha coefficient was 0.79.

#### Data collection

The researcher began sampling women in menopause after obtaining the necessary permits from the management of health centers in Kerman. She did sampling both in person and virtually due to the prevalence of COVID-19. Researchers referred to the health centers of Kerman in the morning for 8 months from January to August 2021 to collect in-person data. First, they explained the study objectives and inclusion criteria. Then, they started sampling after obtaining informed consent and after participants received healthcare services, so they had enough time to answer the questionnaires. Participants completed self-report questionnaires on the demographic information, the use of complementary medicine, and the MRS. The researcher read the questionnaires to uneducated participants. She prepared an electronic questionnaire for online sampling. First, she prepared a list of contact numbers of the women in menopause from 10 health centers, examined their inclusion criteria, and identified the eligible ones. She contacted them and explained the study condition. Furthermore, the researcher explained the study objectives, method, and completion process of the electronic questionnaire to each of the participants, and then asked them to sign the written informed consent form. The link to the questionnaire was sent to eligible postmenopausal women *via* SMS, WhatsApp, and Telegram. The women had enough time to fill out the questionnaires, which were then automatically sent to the researcher.

### Data analysis

SPSS25 was used for data analysis. Descriptive statistics (frequency, percentage, mean, and standard deviation) were used to describe the underlying characteristics of women in menopause, their use of CAM, their satisfaction with CAM, and menopausal symptoms. An independent *t*-test or Mann–Whitney U test was used to compare menopausal symptoms and their dimensions between CAM and non-CAM users. Mann–Whitney U, ANOVA, Kruskal–Wallis, and *t*-tests were used to compare the scores of menopausal symptoms in terms of demographic variables. Multivariate logistic regression was used to determine the predictors of menopausal symptoms. A significance level of 0.05 was considered.

## Results

The mean age of the participants was 54.94 ± 3.08 years (Min = 50 and Max = 60). The mean time passed from the last menstruation was 5.31 ± 3.66 years (Min = 1 and Max = 20). The majority of the participants were educated and unemployed. Only 4.2% of the participants had a history of addiction (opium, cigarettes, or alcohol). About 10.1–49.7% of the participants had a history of diabetes, hypertension, or other chronic diseases (Table 1). In addition, all the participants were married and had no history of uterine cancer or hysterectomy. Only two participants (0.7%) had a history of breast cancer.

**Table 1:** Demographic and clinical information of the participants and their association with menopausal symptoms.

**Variable**	**N (%)**	**Menopausal symptoms**		**Statistical test**	***P*-value**
		**Mean**	**SD**		
**Age (yr.)**					
50–55	159 (55.2)	8.57	5.62	*Z* = −0.22	0.83
56–60	129 (44.8)	8.92	6.68		
**Education level**					
Uneducated	25 (8.7)	7.04	5.05	*H* = 10.90	0.01
Middle/high school	64 (22.2)	10.34	7.05		
Diploma	102 (35.4)	9.34	5.91		
Academic education	97 (33.7)	7.44	5.58		
**Job**					
Unemployed/housewife	202 (70.1)	8.61	6.0	*F* = 3.17	0.04
Employed	36 (12.5)	7.08	5.63		
Retired	50 (17.4)	10.36	6.56		
**Time passed from the last menstruation (yr.)**					
1–2	75 (26.0)	9.61	6.29	*F* = 0.64	0.67
3–4	67 (23.3)	7.85	5.11		
5–6	56 (19.4)	8.89	6.38		
7–8	41 (14.2)	8.66	6.55		
9–10	32 (11.2)	8.22	5.74		
> 10	17 (5.9)	8.82	7.72		
**Children no**.					
0	12 (4.2)	6.58	7.25	*H* = 13.48	0.02
1	27 (9.4)	5.33	3.27		
2	73 (25.3)	8.77	6.26		
3	84 (29.2)	8.86	5.27		
4	49 (17.0)	10.06	7.04		
≥ 5	43 (14.9)	9.60	6.63		
**History of addiction**					
No	276 (95.8)	8.75	6.10	*t* = 0.32	0.75
Yes	12 (4.2)	8.17	6.44		
**History of diabetes**					
No	259 (89.9)	8.78	6.15	*t* = −0.42	0.68
Yes	29 (10.1)	8.28	5.77		
**History of hypertension**					
No	220 (76.4)	8.39	5.81	*Z* = −1.22	0.22
Yes	68 (23.6)	9.82	6.92		
**History of other chronic diseases**					
No	145 (50.3)	8.67	5.88	*t* = −0.16	0.87
Yes	143 (49.7)	8.78	6.35		

SD, Standard Deviation; Z, Mann–Whitney test; H, Kruskal–Wallis test; F, Analysis of variance; t, Independent t-test.

The mean score for the menopausal symptoms of the participants was 8.73 ± 6.11 (Min = 0 and Max = 44). And 28.8% (*n* = 83) of the participants had no/little symptoms, 26.4% (*n* = 76) had mild, 30.6% (*n* = 88) had moderate, and 14.2% (*n* = 41) had severe symptoms. The mean scores for the psychological, somatic-vegetative, and urogenital symptoms are presented in Table 2. In this, 31.6% (*n* = 91) of the participants had no/little psychological symptoms, 27.4% (*n* = 79) had mild, 25.4% (*n* = 73) had moderate, and 15.6% (*n* = 45) had severe symptoms. A 41.7% (*n* = 120) of the participants had no/little somatic-vegetative symptoms, 27.8% (*n* = 80) had mild, 24.7% (*n* = 71) had moderate, and 5.8% (*n* = 17) had severe symptoms; 33.7% (*n* = 97) of the participants had no/little urogenital symptoms, 25.3% (*n* = 73) had mild, 21.2% (*n* = 61) had moderate, and 19.8% (*n* = 57) had severe symptoms.

**Table 2:** Comparison of menopause symptoms in CAM users and non-users.

**Variable**	**Mean (SD)**	**CAM User**		**Statistical test**	***P*-value**	**Effect size**
		**Yes (Mean/SD)**	**No (Mean/SD)**			
Psychological domain	3.40 (2.97)	3.59 (3.01)	3.06 (2.87)	*t* = −1.43	0.15	0.18
Somatic-vegetative domain	3.56 (2.67)	3.99 (2.78)	2.70 (2.21)	*Z* = −3.90	<0.001	0.51
Urogenital domain	1.77 (2.0)	2.08 (2.12)	1.17 (1.60)	*Z* = −3.67	<0.001	0.48
Total score	8.73 (6.11)	9.66 (6.20)	6.96 (5.55)	*t* = −3.66	<0.001	0.46

SD, Standard Deviation; CAM, Complementary and Alternative Medicine; t, Independent t-test; Z, Mann–Whitney test.

About 65.3% (*n* = 188, 95% confidence interval = 59.7–71.2) of the participants reported using at least one type of CAM in the previous year. The frequency of using each type of CAM is presented in Table 3. Here, 60.6% (*n* = 20), 59.15% (*n* = 84), and 31.76% (*n* = 27) used nutritional supplements, prayer, and medicinal herbs once a day, respectively; 22.73% (*n* = 5), 18.18% (*n* = 4), and 14.81% (*n* = 4) used massage, wet cupping, and dry cupping once a month, respectively. In addition, 61.54% (*n* = 32) used relaxation and meditation more than once a week. The mean score of satisfaction with using CAMs was 21.60 ± 5.87 (Min = 0 and Max = 36), which was higher than the midpoint of the scale, that is, 18.

**Table 3:** The use of different methods of CAMs and its association with menopause symptoms.

**Variable**	**Frequency of**	**95% Confidence**	**Menopause**		**Statistical test**	***P*-value**
	**the users (%)**	**interval of percentage (%)**	**symptoms**			
			**Mean**	**SD**		
**Medicinal herbs**						
No	203 (70.5)	65.3–75.7	7.89	5.99	*t* = −3.68	**<** **0.001**
Yes	85 (29.5)	24.3–34.7	10.73	5.95		
**Dry cupping**						
No	261 (90.6)	87.2–93.4	8.46	6.07	*t* = −2.28	**0.02**
Yes	27 (9.4)	6.6–12.8	11.26	6.01		
**Wet cupping**						
No	266 (92.4)	89.2–95.5	8.81	6.15	*t* = 0.83	0.40
Yes	22 (7.6)	4.5–10.8	7.68	5.58		
**Massage**						
No	266 (92.4)	89.2–95.5	8.68	6.0	*t* = −0.4	0.69
Yes	22 (7.6)	4.5–10.8	9.23	7.40		
**Nutritional supplements**						
No	255 (88.5)	84.7–92.0	8.67	6.0	*t* = −0.42	0.67
Yes	33 (11.5)	8.0–15.3	9.15	6.96		
**Relaxation and meditation**						
No	236 (81.9)	77.1–86.1	8.34	6.10	*t* = −2.31	**0.02**
Yes	52 (18.1)	13.9–22.9	10.48	5.87		
**Prayer**						
No	146 (50.7)	44.8–56.3	7.63	5.91	*t* =-3.13	**0.002**
Yes	142 (49.3)	43.8–55.2	9.85	6.12		
**Other**						
No	282 (97.9)	96.2–99.3	8.62	6.04	*t* = −2.08	**0.04**
Yes	6 (2.1)	0.7–3.8	13.83	7.52		
**Number of CAMs methods**						
0	100 (34.7)	29.2–40.3	6.96	5.55	*H* = 19.98	**0.001**
1	68 (23.6)	18.4–28.5	8.76	6.44		
2	67 (23.3)	18.4–28.1	9.76	6.23		
3	34 (11.8)	8.3–15.6	10.44	4.62		
≥4	19 (6.6)	3.8–9.7	11.16	7.51		

CAM, Complementary and Alternative Medicine; SD, Standard Deviation; t, Independent t-test; H, Kruskal–Wallis test, Other: Acupressure, acupuncture, and homeopathy.

There were significant differences in menopausal symptoms, somatic-vegetative, and urogenital domains between the CAM-users and non-CAM users (*p* < 0.001) (Table 2). In addition, there was a significant association between medicinal herbs (*p* < 0.001), dry cupping (*p* = 0.02), relaxation and meditation (*p* = 0.02), prayer (*p* = 0.002), and other methods (*p* = 0.04), and menopausal symptoms (Table 3).

Among the other study variables, there was a significant association between educational level (*p* = 0.01), job (*p* = 0.04), number of children (*p* = 0.02), and menopausal symptoms (Table 1). A multivariate logistic regression model with a backward method was used for further analysis. The independent variable was the menopausal symptoms (yes/no), while the dependent variables were educational level, job, number of children, and CAM user. The results showed an association only between CAM users and menopausal symptoms. On the other hand, women with menopausal symptoms used more CAM methods compared with women without menopausal symptoms (Odds ratio = 2.25, 95% confidence interval = 1.33–3.80).

## Discussion

The study results showed that the menopausal symptoms were mild and desirable, with 28.8% of the women having no menopausal symptoms, while 14.2% of them had severe menopausal symptoms. The mean score of the somatic-vegetative domain was high, which included symptoms such as sexual dysfunction, vaginal dryness, and urinary symptoms. Bahri et al. (53) in Iran also showed a desirable level of menopausal symptoms. According to Rathnayake et al. (54) in Sri Lanka, 47.6% of the women in the menopausal stage reported severe to very severe musculoskeletal disorders, which was inconsistent with the present study. Masjoudi et al. (48) in Iran found that 43.8% of the women in the menopausal stage were asymptomatic, 55.2% experienced mild to severe menopausal symptoms, and 1% experienced moderate to severe menopausal symptoms, which was inconsistent with the present study. Jalili et al. (55) in Iran reported that moderate, severe, and very severe menopausal symptoms were related to sleep disorders, muscle pain, and hot flashes, respectively, which contradicted the present study. According to Makvandi et al. (56) in Iran, the total score of menopausal symptoms was moderate, and the most common menopausal symptoms were muscle pain, sleep problems, and memory loss, which contradicted the results of the present study. This study was different from that of Makvandi et al., Masjoudi et al., Jalili et al., and Rathnayake et al. due to participants' ages, ethnic differences between the studied cities, and geographical, individual, and cultural differences between the studied countries, respectively (56). Menopause exposes women to extensive changes caused by low estrogen levels, including hot flashes and night sweats, dizziness, irregular and rapid heartbeat, atrophy of the vaginal mucosa, sleep disturbance, musculoskeletal pain, difficulty concentrating and memory disorders, and despair (2, 57–59).

The study results also showed that 65.3% of the participants used at least one type of complementary and alternative medicine in the last year, and 60.60, 59.15, and 31.76% of people used dietary supplements, prayers, and medicinal herbs once a day, respectively. According to Ozcan et al. (15) the majority of Turkish women used herbal medicines, dietary supplements, mind-body exercises, and religious approaches to combat hot flashes. Arentz et al. (16) found that more than 70% of Australian women with polycystic ovary syndrome used dietary and herbal supplements. However, Witteman et al. (60) showed that 68.7% reported using CAM/self-care strategies to treat the symptoms of vaginal infection. The types and patterns of CAM are related to socio-cultural backgrounds and are generally influenced by culture, history, and sometimes religion (51). The studies inconsistent with the present one had differences in the sample size, research tools, and cultural and religious factors. The majority of Iranian people are Muslims, who pray every day, and religion and spirituality are tied to their lives (51).

The study results showed that the severity of menopausal symptoms and scores in somatic-vegetative and urogenital domains were higher in CAM users compared to non-CAM users. Women experiencing menopausal symptoms used complementary and alternative medicine more than other postmenopausal non-CAM users. However, the study results indicate no significant difference in the psychological domain between CAM and non-CAM users. According to the study results, there was a statistically significant difference in the severity of menopausal symptoms, medicinal herbs, dry cupping, relaxation and meditation, prayer, and other methods between CAM and non-CAM users. The study results showed that women in the menopausal stage suffered from vasomotor symptoms, psychological symptoms, musculoskeletal pain, osteoporosis, sleep problems, and genital symptoms. Menopause is very stressful for some women, so stress management strategies, such as relaxation, biofeedback, aerobics, yoga, meditation, and breathing techniques can help them cope with menopause and aging in general, and improve their menopausal symptoms (61). According to Ozcan et al. (15) in Turkey, the majority of CAM users in menopause reported lower severity of hot flashes compared with other women in menopause. The results of an evidence-based systematic review study showed that licorice, valerian, soy, sage, ginseng, and so on, improved menopausal symptoms (62). Kheirkhah and Firooznia in Iran also found that herbal tea capsules, Passion flower, Vitex agnus-castus, Liquorice, and soy improved hot flashes among women in menopause (63, 64). Taavoni et al. (65) in Iran showed that Lemon Balm reduced physical, psychological, and reproductive symptoms in postmenopausal women. The studies mentioned above contradicted the present study, in which women who used medicinal herbs experienced more menopausal symptoms. This discrepancy can be attributed to differences in research type, age, and research tools. Johnson et al. (66) showed that mind-body exercise might help reduce stress and some menopausal symptoms among women, and hypnosis clinically had a significant effect on reducing hot flashes. Their result was inconsistent with that of the present study, in which users of yoga had a high level of menopausal symptoms. According to the present study, dietary supplements did not affect the severity of menopausal symptoms. Therefore, Jokar et al. (67) in Iran showed that vitamin C was not effective in reducing depression among postmenopausal women. They recommended further studies on the effect of vitamin C on anxiety and depression among postmenopausal women. Shafiee et al. (28) indicated that spiritual interventions were effective in reducing depression and improving the psychological dimension among postmenopausal women. Their study was inconsistent with the present one regarding the psychological dimension. The present study showed the effectiveness of acupressure in improving menopausal symptoms, but according to Armand et al. and Abedian et al. acupressure was effective in reducing anxiety, hot flashes, night sweats (68), and sleep disorders (69). Sultana in India found that dry cupping was effective in reducing the pain intensity of dysmenorrhea, which was inconsistent with the present study (70). The differences among recent studies may be due to the differences in research tools, the type of intervention, and sample size, as well as personal, cultural, and geographical differences.

## Limitations

Self-assessment was used to collect the data, so the menopausal symptoms might be reported less realistically. Therefore, a female researcher gathered information to overcome this limitation so that the participants felt less ashamed and upset. Postmenopausal women were rarely referred to the health centers during the COVID-19 outbreak. To overcome this limitation, a combination of face-to-face and virtual sampling was used. Postmenopausal women did not know about some complementary and alternative medicines, so the researchers tried to give them information about the types of complementary medicine. Another limitation was that postmenopausal women were selected from only one city. Performing national and international studies makes it possible to generalize these results. In addition, the small number of female researchers was one of the limitations of the present study.

## Conclusion

According to the study results, 30.6% of the women suffered from menopausal symptoms moderately, while 14.2% of them suffered from menopausal symptoms severely. The total score of menopausal symptoms and the scores of somatic-vegetative and urogenital domains were higher in CAM users compared with non-CAM users. However, the study results indicated no significant difference in the psychological domain between CAM and non-CAM users. There was also a statistically significant difference in the severity of menopausal symptoms between users and non-users of the medicinal herbs, dry cupping, relaxation and meditation, prayer, and other methods. According to the study results as well as the severity of menopausal symptoms in women who used complementary medicine, more studies are suggested to investigate the use of complementary and alternative medicine among postmenopausal women. In addition, further studies are recommended to investigate the effects of complementary and alternative medicine on the psychological domain to relieve anxiety, stress, depression, and sleep disorders among women. Given that the present study was performed on women aged 50–60 years who were in normal menopause, it is recommended that other studies be conducted to evaluate the effect of complementary medicine on the severity of menopause in women aged above 60 years who were not in normal menopause.

## Data availability statement

The raw data supporting the conclusions of this article will be made available by the authors, without undue reservation.

## Ethics statement

The studies involving human participants were reviewed and approved by Kerman University of Medical Sciences. The patients/participants provided their written informed consent to participate in this study.

## Author contributions

MD designed the study. MD, ZI, and AGH wrote the manuscript. MD and AGH contributed to the study design, provided critical feedback on the study and statistical analysis, and inputted the draft of this manuscript. ZI and MHA collected data. All authors have read and approved the final manuscript.

## Conflict of interest

The authors declare that the research was conducted in the absence of any commercial or financial relationships that could be construed as a potential conflict of interest.

## Publisher's note

All claims expressed in this article are solely those of the authors and do not necessarily represent those of their affiliated organizations, or those of the publisher, the editors and the reviewers. Any product that may be evaluated in this article, or claim that may be made by its manufacturer, is not guaranteed or endorsed by the publisher.
